# Inter-relationships between objective handwriting features and executive control among children with developmental dysgraphia

**DOI:** 10.1371/journal.pone.0196098

**Published:** 2018-04-24

**Authors:** Sara Rosenblum

**Affiliations:** Laboratory of Complex Human Activity and Participation (CHAP), Department of Occupational Therapy, Faculty of Welfare and Health Sciences, University of Haifa, Mount Carmel, Israel; Kyoto University, JAPAN

## Abstract

**Objective:**

To describe handwriting and executive control features and their inter-relationships among children with developmental dysgraphia, in comparison to controls.

**Method:**

Participants included 64 children, aged 10–12 years, 32 with dysgraphia based on the Handwriting Proficiency Screening Questionnaire (HPSQ) and 32 matched controls. Children copied a paragraph onto paper affixed to a digitizer that supplied handwriting process objective measures (Computerized Penmanship Evaluation Tool (ComPET). Their written product was evaluated by the Hebrew Handwriting Evaluation (HHE). Parents completed the Behavior Rating Inventory of Executive Function (BRIEF) questionnaire about their child's executive control abilities.

**Results:**

Significant group differences were found for handwriting performance measures (HHE and ComPET) and executive control domains (BRIEF). Based on one discriminate function, including handwriting performance and executive control measures, 98.4% of the participants were correctly classified into groups. Significant correlations were found in each group between working memory and legibility as well as for other executive domains and handwriting measures. Furthermore, twenty percent of the variability of the mean pressure applied towards the writing surface among children with was explained by their 'emotional control' (BRIEF).

**Conclusion:**

The results strongly suggest consideration of executive control domains to obtain better insight into handwriting impairment characteristics among children with dysgraphia to improve their identification, evaluation and the intervention process.

## Introduction

Handwriting still serves as the most immediate form of graphic communication, despite the expanding use of technology [1]. Skilled handwriting is essential for school-aged children. This skill allows them to write within a reasonable amount of time and to create a readable product through which thoughts and ideas can be communicated [2].

Children typically acquire skillful handwriting performance during the first three years of school. With this skill they are able to automatically write a legible product while keeping in line with the expected time demands of the class schedule [2, 3]. Though, previous research has established that a large number of children do not yet write automatically by this age. These children are either diagnosed with dysgraphia or need to cope with ongoing difficulties with handwriting [4]. In the Diagnostic and Statistical Manual of Mental Disorders (5^th^ ed.; DSM–5) [5] dysgraphia is coded as a "Specific Learning Disorder with impairment in written expression" [5]. However, no specific diagnosis criteria are provided. The current study followed Hamstra-Bletz and Blote's [6] definition that describes dysgraphia as a disturbance or difficulty in the production of written language related to the mechanics of writing. The inadequate handwriting performance is seen among children who have at least an average intelligence level and who have not been diagnosed with any apparent neurological or perceptual-motor difficulties.

Despite evidence of dysgraphia among 10% to 34% of all school-aged children [7, 8], research on developmental dysgraphia is sparse [9]. Fine motor activities and predominantly writing tasks compose of 30% to 60% of an average school day [10]. Therefore, handwriting deficits as such or dysgraphia can harm children’s confidence and self-image, and consequently affect their academic achievements [11, 12].

Handwriting is a complex activity that entails an intricate blend of cognitive, kinesthetic, and perceptual–motor components [1]. In order to produce a hand-written product, the child needs to simultaneously activate sensory-motor and cognitive skills, devise an idea, plan the structure of the sentence syntax and spelling, attain motor-orthographic integration to create the text, and to appraise the obtained result [13, 14].

Although previous studies have searched for the underlying mechanism behind the phenomena of writing difficulties (e,g, [15, 16]), more information is still essential to improve the theoretical knowledge about the phenomena and to develop appropriate evaluation and intervention methods.

As part of the process required for producing the written output, the current study focused on the "transcription phase" which is considered the “lower level” of writing production [17]. This differs from the generation of the written content and ideas, which are considered to be the “higher level” of the writing process. The transcription phase includes the processes involved in retrieving letterforms and spelling of familiar words from the long-term memory, strategically spelling new and unfamiliar words. This stage also includes motor planning that enables the hand movements required to graphically generate the letters.

Despite this phase being labeled as “lower level,” the complexity of this production phase is evident in the literature. Founded on various handwriting models (e.g., [16, 18–21] as described in detail previously [15], handwriting transcription can be depicted as a hierarchically organized representation of mental motor movements. These models premise that handwriting occurs due to distinct processing activities whereby the output from an earlier stage forms the input for the next stage.

More specifically, as described in Denckla and Roeltgen's [18] model, and further emphasized by Graham and his colleagues, [22], handwriting production involves a group of higher-level cognitive defined as executive functions (EFs). These cognitive skills enable the organization of one's behavior they operate and oversee the person's perceptions, feelings, thoughts and actions. EF operate as a group of “co-conductors” that direct people to achieve successful goal-directed behavior [23, 24]. Skills encompassed in EF include high-level cognitive functions such as setting and managing goals, planning, organization, inhibition, coping with complexity, shifting among different cognitive situations and requirements, working memory and metacognition [24, 25]. McCloskey et al. [23] linked executive deficits with handwriting production features. They claimed that difficulties in text formation, poor text production, speed and automaticity can result from disuse, ineffective or inconsistent use of EF [23].

While literature regarding the relationships between EF and linguistic and content aspects of writing exits [22, 26–28], literature is scarce concerning the relationships between EF deficits and handwriting performance, such as the transcription lower level phase. Consequently, it is essential to consider which tools should be used to measure both EF and handwriting in a way that can reflect real-life daily function [2, 29]. Researchers have stated that evaluation performed by a collection of tests, which represent separate components of EF is not ecologically valid and is insufficient [30, 31], because these tests do not reflect the complex multifactorial nature of everyday functioning [32]. Therefore, in the current study EF and handwriting was evaluated in the context of real-life daily function.

Literature about the relationships between EF as measured through daily function and handwriting process and product features specifically among children with developmental dysgraphia is absent. Results of previous research indicated that executive abilities, as expressed in daily organization in space and time among children with dysgraphia, were significantly inferior compared to the abilities of controls [33]. Organizational deficits as such were also found among children and adults with developmental coordination disorders (DCD) confronted with handwriting difficulties [15, 34, 35]. Furthermore, relationships were found between their daily organizational abilities and specific handwriting process and product measures (for more details see: [15, 34, 35].

Thus, this two-phased study aimed to obtain better insight into the handwriting process and product features of children aged 10–12 with dysgraphia, their behavioral daily manifestations of EF abilities and the relationship between these domains. In the first phase, the differences between children with dysgraphia and those with typical development (TD) were analyzed. In the second phase, deeper analysis among the children with TD and those with dysgraphia was performed, and the research hypotheses were: 1. Children with dysgraphia aged 10–12 will differ from typical peers in their handwriting performance characteristics as measured by the Computerized Penmanship Evaluation Tool (ComPET) and by the Hebrew Handwriting Evaluation (HHE). 2. Significant group differences will be found for their EF. 3. Specific handwriting measures/scores and EF will best discriminate between school-age children with TD and those with dysgraphia. 4. Significant correlations will be found between certain handwriting performance characteristics and EF domains among children with TD and those with dysgraphia and 5. Handwriting performance as screened by the teacher will predict EF domains while those executive domains will predict the variation of handwriting process and product measures among the children in each group.

## Methods

### Participants

Sixty-four children aged 10–12 years old were recruited from eight schools in the northern district of the country. Thirty-two children were defined by their teacher as having dysgraphia based on the Handwriting Proficiency Screening Questionnaire (HPSQ) [36], and 32 were age and gender matched controls with TD. Children with known neurotic/emotional disorders, autistic disorders, physical disabilities, neurological diseases, attention deficit hyperactivity disorder (ADHD) or dyslexia, were excluded from the study. All children were native Hebrew speakers and writers (for at least 4 years), who attended school, and reported no hearing or vision problems. For each child with dysgraphia, a matched control from same school and class was recruited. There were no significant differences between the groups for gender (24 boys, 8 girls in each group) nor for age (dysgraphia group: M = 11.16±7.00; control group: M = 11.25±6.5).

### Instruments

Handwriting Proficiency Screening Questionnaire (HPSQ) [36]. The HPSQ is a ten-item, reliable and valid questionnaire developed to identify school-aged children with impairment in written expression [5], based on their teacher’s observation.

The 10 items encompass the most vital indicators of handwriting impairment according to the definition of dysgraphia suggested by Hamstra-Bletz and Blote [6] including legibility, performance time and physical and emotional well-being. Items are scored on a 5-point Likert scale from 0-never to 4-always, and then summed to a final score. Examples of items in each indicator: 1. Legibility: Is the child's writing unreadable? 2. Time: Does the child not have enough time to copy tasks from the blackboard? 3. Physical and emotional well -being: Does the child complain about pain while writing? Cut-off scores were determined for handwriting impairment as equivalent or above 14 points [36]. In the current study the Cronbach Alpha reliability of the legibility items (1,2,10) was α = .88, the time items (3,4,9) was α = .92 and the well-being items (5–8) was α = .89.

Handwriting product evaluation-The Hebrew Handwriting Evaluation (HHE) [37] and Computerized Penmanship Evaluation Tool (ComPET, previously referred to as POET) [38]: Children copied a paragraph onto a sheet of paper affixed to a Wacom Intous II x-y digitizing tablet (404 X 306 X 10 mm), while using a wireless electronic pen with a pressure-sensitive tip (Model GP-110). This constitutes part of the ComPET system that enables receipt of exact time of task performance in seconds, the mean pressure applied towards the writing surface in non-scaled units from 0–1024 and mean strokes height which reflects the letter's height in millimeters. The HHE is a valid and reliable tool used in this study to collect children’s written product data. The HHE includes global and analytic outcome measures of the written product. The global legibility (the overall clarity) is scored on a 4-point Likert scale (from 1-most legible to 4-least legible). The analytic measurement of legibility used in the HHE examined three variables: a. number of letters erased and/or written over. b. number of unrecognizable letters due to the quality of letter closure, rounding of letters, or letter reversals. c. spatial arrangement of the written text, including vertical alignment of letters on the line, the spacing of words and letters and letter size. The spatial arrangement scores range from 6 (best performance) to 24 (worst performance). Furthermore, the number of letters written in the first minute is recorded.

Behavioral Rating Inventory of Executive Function (BRIEF) [39]: A parental report and a reliable and valid questionnaire used to assess daily behavioral manifestations of EF deficits in 5-18-year-olds. Eighty-four statements are divided into eight domains (inhibition, shifting, emotional control, initiation, working memory, planning/organization, organization of materials, and monitoring). Parents rate the frequency of the behavior described in each statement from 1-low to 3-high. Domain scores are summed into two index scores and combined into an overall Global Executive Composite. A standard score of 65 and above indicates a deficit [39]. In the current study, only the eight domain scores were used in the statistical analysis.

### Procedure

Ethical approval to perform this study was obtained from the Ministry of Education and Culture, Northern Region and the University of Haifa Ethics Committee (No. 029/06). Children suspected as having dysgraphia were identified by their teacher by means of the HPSQ scale and parents provided signed consent. For each child in the dysgraphic group, a matched control child without dysgraphia was chosen and both the child and parents signed their consent. Children then performed the paragraph copying task on the digitizer. The children's written product was evaluated by an occupational therapist at the school, who was blinded to their HPSQ scores. Parents completed the BRIEF questionnaire about their child.

### Data analysis

Data was analyzed using the SPSS for Windows analysis program (SPSS, Inc.). Descriptive statistics of the dependent variables were tabulated and examined. Chi square and t-tests were conducted to compare differences in the children's gender, age, HPSQ scores and kinematic measures of handwriting performance (ComPET) in both groups. To examine whether the children with dysgraphia differed with respect to handwriting product scores, frequencies and Mann-Whitney analyses were performed. MANOVA analyses were then used to test for group differences across the BRIEF domains. Univariate ANOVA analyses were used to determine the source for the group differences. Discriminant analysis was conducted in order to determine which of the handwriting product (HHE), process measures (ComPET) and EF domains scores (BRIEF) were the best predictors of group membership.

To investigate the relationships between the BRIEF domains and handwriting product (HHE) and process (ComPET) measures among the children with TD and children with dysgraphia, Spearman and Pearson correlational analyses were performed, respectively. Three stepwise multiple regressions were applied in each group in order to determine: 1. Which of the HPSQ handwriting performance scores (legibility, time and well-being) add to the prediction of EF (BRIEF) domains, 2. Which of the EF domains add to the prediction of the mean pressure applied to the writing surface, and 3. Mean pen strokes height (ComPET).

## Results

### Differences between groups in Handwriting Proficiency Screening Questionnaire (HPSQ) scores

As expected, due to HPSQ-based selection of children with dysgraphia, significant between group differences were found for the HPSQ mean final score (TD: 1.03±1.79; dysgraphic: 21.21±2.90 *t*(62) = 33.58 *p* < .0001).

#### Differences between groups’ handwriting performance measures (product HHE and process, ComPET)

The two measures that reflect performance time, as measured by the HHE, by the *number of letters written in the first minute*, and the *total performance time* (ComPET), indicated significant group differences with a considerable gap between the groups (see Table 1). Results of the Mann Whitney U-test on the other four HHE outcome measures yielded significant differences for all HHE products' outcome measures. No significant group differences were found for the pressure applied to the writing surface, though significant differences were found for mean pen stroke height.

**Table 1:** Comparison between groups' handwriting performance measures, HHE and ComPET.

HHE	Children with TD (n = 32) M ± SD	Children with Dysgraphia (n = 32) M ± SD	t/u
			U
Global Legibility	1.40 (.61)	2.43 (.62)	150.00***
No. of Letters Erased or over-written	.78 (1.00)	3.37 (1.68)	85.00***
Unrecognizable Letters	4.50 (4.10)	9.87 (6.32)	231.50***
Spatial Arrangement	7.84 (2.03)	11.28 (2.09)	111.00***
Number of letters written in the first minute	66.71 (13.07)	47.12 (13.05)	159.00***
**ComPET**			
Total performance time (s')	162.13 (29.38)	219.97 (48.00)	t(62) = 5.81***
Mean pressure (0–1024)	693.55 (131.52)	656.90 (137.78)	t(62) = -1.01 NS
Mean strokes height (m'm)	3.19 (.64)	3.63 (.94)	t(62) = 2.16 *

*p < .05

***p≤.000

#### Differences between groups in executive control (BRIEF scores)

The MANOVA analyses testing for group differences (TD versus dysgraphic) across the eight BRIEF domains yielded significant differences between the groups (*F*_(8,55)_ = 26.28; *p* < .0001 η2_p_ = .79). Following ANOVAs indicated significant differences between the groups in each of the eight executive control domains with high effect sizes (see Table 2). The significant differences persisted after applying a Bonferroni correction (.05/8 = .006).

**Table 2:** Comparison of the Behavioral Rating Inventory of Executive Function (BRIEF) scores between groups.

BRIEF	Children with TD (n = 32)	Children with Dysgraphia (n = 32)	F(1,62)	η^2^
	M (SD)	M (SD)		
Inhibition	45.53 (5.97)	49.65 (9.75)	13.49***	.18
Shift	50.09 (8.14)	66.34 (10.36)	48.63***	.44
Emotional control	45.40 (7.21)	56.03 (10.26	22.95***	.27
Initiative	43.81 (6.73)	59.78 (8.77)	66.63***	.52
Working memory	43.06 (5.00)	62.25 (6.34)	180.45***	.74
Planning/organization	42.84 (5.11)	62.84 (8.25)	142.25***	.69
Organization of materials	44.90 (6.16)	57.31 (7.77)	50.06***	.44
Monitoring	41.25 (6.32)	59.18 (7.27)	110.79***	.64

***p < .001

#### Group discrimination based on handwriting performance (HHE and ComPET) and EF control (BRIEF)

Discriminate analysis was conducted to define which of the eight BRIEF domains and the continuous handwriting performance measures is effective in predicting category membership (see Table 3). One discriminate function was found for group classification of all participants (Wilks' Lambda = .14, *p* < .0001). Working memory made the greatest contribution to group membership (.697), while the planning/organization was .618 and monitoring was .546. Based on this function, 98.4% of the participants overall, 100% of the dysgraphic and 96.9% of the controls were correctly classified. A Kappa value of .969 (*p* < .0001) was calculated, demonstrating that group classification did not occur by chance.

**Table 3:** Discriminant analysis structure matrix predictors' loading values.

	Loading value		Loading value
Working memory (BRIEF)	.697	Shift (BRIEF)	.362
Plan/organize (BRIEF)	.618	Total performance time (ComPET)	.301
Monitoring (BRIEF)	.546	Emotional control (BRIEF)	.248
Initiation (BRIEF)	.423	Inhibition (BRIEF)	.191
Letters erased or written over (HHE)	.388	Mean stroke's height (ComPET)	.112
Organization of materials (BRIEF)	.367	Mean pressure (ComPET)	-.056

#### Correlations between handwriting performance characteristics (HHE and ComPET) and EF control domains (BRIEF) among children with TD and among children with dysgraphia

As presented in Table 4, significant correlations were found in both groups between working memory and handwriting legibility (HPSQ) (*r* = .35; .36 *p* < .05). In the TD group, working memory was also significantly correlated with the number of letters erased or over-written while writing (HHE) (*r* = .58 *p* < .01) and initiation was significantly correlated with the mean pen stroke height (*r* = -.35 *p* < .05).

**Table 4:** Correlations between the HPSQ scores and executive control domains among children with TD (*n* = 32) (regular font) and among children with Dysgraphia (*n* = 32) (underlined).

	Executive control: BRIEF							
	Inhibit	Shift	Emotional Control	Initiate	Working Memory	Plan/ Organize	Organization of Materials	Monitoring
**HPSQ**								
Legibility					.36*			
					.35*			
Time								
Well- being								
**HHE**								
Number of erasements					.58**			
**ComPET**								
Total time (seconds)								
Pressure (0–1024)	.44**		.45**		.40*			.39*
Mean strokes height (mm)				-.35*				.44*

* p < .05

**p < .01

Among the children with dysgraphia, inhibition, emotional control, working memory and monitoring significantly correlated with the mean writing pressure (*r* ranges .39-.45 *p* < .05-.01). Furthermore, monitoring significantly correlated with their mean stroke height (*r* = .44 *p* < .05).

Following the discriminant analysis results and the altered correlations found significant in the two groups, the regression analysis was performed separately in each group.

#### Predictability of mean handwriting pressure applied to the paper (ComPET) by EF domains (BRIEF) in each group (Children with TD versus children with dysgraphia)

The five EF domains (working memory, initiation, inhibition, emotional control and monitoring) were entered as possible predictors of *mean pressure* applied towards the writing surface in each group. None of the EF domains predicted mean pressure among the children with TD. In the group of children with dysgraphia, emotional control predicted 20% of the mean pressure applied to the writing surface, *F*_(1,30)_ = 7.94 β = .46 *p* < .001.

#### Predictability of mean stroke height (ComPET) by EF domains (BRIEF) among each group (Children with TD versus children with dysgraphia)

Among children with TD, as presented in Table 5, initiation accounted for 12% of the variance to prediction of mean stroke's height, *F*_(1,30)_ = 4.28 β = -.35, *p* = .047, while organization of materials added 18% to the prediction of strokes height among this group, *F*(1,29) = 7.33 β = -.59 *p* = .011. All in all, the two EF executive control domains accounted for 30% of the variance in the number of mean stroke's height among children with TD.

**Table 5:** Predicting mean stroke height by EF domains (BRIEF) among TD group.

	Model 1			Model 2		
Variable	B	SE B	β	B	SE B	β
Initiation	-.34	.02	-.35*	-.06	.02	-.59**
Organization of materials				.05	.02	.48**
R^**2**^ (Adj.rsq)	.12			.30		
F change in R^**2**^	4.28*			7.33*		

*p < .05

**p < .01

Interestingly, a different picture was received among children with dysgraphia, where 17% the variance of *strokes mean height* was predicted by monitoring, *F*_(1,30)_ = 7.39, β = .44, *p* < .05.

## Discussion

The aim of this study was to describe handwriting and EF features and their inter-relationships among children with developmental dysgraphia in comparison to controls. Similar to previous studies, results of the current study indicate that children who were defined by their teachers as having dysgraphia based on the HPSQ indeed showed significantly inferior writing abilities related to performance time and global legibility [1]. Furthermore, their letters were significantly higher. Developmentally, it has been established that as a child grows, there is a decrease in letter size [6, 40]. This decrease is a result of more developed motor control in the distal areas of the hand and wrist, thus enabling the performance of hierarchical and sequential smaller movements [41]. Based on previous findings about their handwriting production features (e.g., [33, 42, 43], it seems that children with dysgraphia do not develop appropriate control of the writing tool which would enable them to reduce the letter size and produce a legible text, as children in the control group were able to do.

The results which indicated significantly less organization and more letters erased/overwritten and unrecognizable letters among children with dysgraphia are similar to previous results found among children aged 8–9 with dysgraphia [2, 42]. Thus, the pattern previously demonstrated among younger children with dysgraphia of unsmooth handwriting with more erasures and less efficient writing speed [8], was also exhibited in the current study among children aged 10–12 years.

While focusing on possible underlying mechanisms of deficient performance, the results indicated that the BRIEF scores of children with dysgraphia were significantly higher than those of controls, thus indicating lower EF control. Although the children with dysgraphia did not achieve a score of 65, which indicates deficit EF according to the BRIEF manual [39], it is interesting to note the significant difference between their EF as reflected by the BRIEF scores compared to those of the TD group. Findings which do not indicate EF deficits according to the BRIEF, yet present significant differences between children with TD have also been found among children with other neurodevelopmental disabilities. Examples of these neurodevelopmental disabilities are ADHD [44], Specific Language Impairments [45], as well as among children with Traumatic Brain Injury [46]. It is important to note that all the above-mentioned disabilities and not only dysgraphia are tied with daily function deficits at home and at school. The significant differences in the BRIEF results may express that these children can have significant difficulties in daily life situations but obtain within norm scores on standardized tests that are administered in a profoundly structured assessment environment [47]. Thus, further studies are required to continue to analyze the meaning of such BRIEF scores which do not fall into the clinically impaired range, but significantly differ from those of children with TD in relation to children's daily function, including handwriting.

Although there is no previous clear evidence in the literature, based on the models and literature described in the introduction, EF are involved in the transcription phase of handwriting [16, 18, 20]. In fact, efficient handwriting transcription performance requires shifting, working memory, planning and organization, monitoring and material organization, which are components of EF. Therefore, it is not surprising that those EF domains did not only significantly differ among children with dysgraphia but also had the highest effect size among the EF domains.

Indeed, results of the correlations and discriminant analysis indicated that some of the EF domains scores were related and well contributed to group differentiation. Working memory was the domain with the highest loading value, while planning and organization of materials, monitoring and initiation EF domains were further on. The importance of working memory and other EF domains has been stressed in previous studies related to the written content such as language production, formulation of ideas and linguistic expression (e.g., [26, 28, 48]. Indeed, results of the current study indicated the significant moderate correlation found between working memory and the number of letters erased or overwritten among the children with TD. However, a different picture was found among the children with dysgraphia. Apart from the fact that not only writing legibility but also handwriting well-being was predicted by working memory in this group, the dissimilar correlations and regression analysis results in each of the groups strengthen the impression that a different underline mechanism of the handwriting performance possibly occurs in each group.

When gathering together the results of the children with dysgraphia to create one complete picture, two important findings were revealed. Firstly, besides working memory, scores of inhibition, emotional control, and monitoring also significantly moderately correlated with the *mean pressure* they applied towards the writing surface. Secondly, the emotional control of the children with dysgraphia predicted 20% of the variance of the applied pressure, while none of the EF domains predicted the applied pressure among children with TD. Thirdly, besides the prediction of pressure by the emotional control, 17% of the mean pen strokes height was predicted by monitoring ability in this group.

It seems that children with dysgraphia are busy thinking and planning how to produce the letters in space and time due to their deficient ability to initiate, plan, organize and monitor the letters/words on the paper. In fact, these difficulties accompany the children with dysgraphia in their daily performance in varied domains as reported by their parents and as manifested through the significant differences in BRIEF scores. More studies are needed to discover whether the applied pressure and controlling pen strokes height while writing reflect these children's self-regulation ability which is critical for varied domains in daily life besides handwriting [49].

Volman and colleagues [16] identified deficient cognitive planning ability and handwriting difficulties among children aged 7.5–9.5 in grades 2 and 3 [16]. However, to our knowledge, the relations between EF domains and writing production among children aged 10–12 years, whose handwriting production is expected to be skilled, mature and automatic from 8–9 years of age [1], have not yet been studied. As far as is known, this is the first evidence of the relationships that occur between the need to control the letters production and emotional stress involved in the process in this group of children.

These results may have clinical implications as they provide insight into the amount of energy that these children need to invest in cognitive and emotional resources. While children need to invest energy in orthographic motor processing, such as organization of letter height/forms as well as their location on the page, they are unavailable to think about and plan the writing content [20]. This may also be linked with fatigue and frustration [50]. Such results raise questions about how to improve their performance in a way that will allow them to preserve their resources for the other tasks in class and at home.

In fact, the results reflect the ongoing dance between the higher and lower level cognitive process as described by Volman et al. [16]. The current results present the relationships between EF and the deficits in the triangular control ability of time, space and force required while writing among children with dysgraphia, as reflected by objective measures of the process [51]. This deficient control was previously exhibited in the variability of the created pen strokes [42]. Such results may imply that cognitive-emotional relationships occur in real life performance, and as far as is known, this is the first time that such possible relationships have been measured through objective kinematic measures of real performance.

Over the years, although clinicians have considered the amount of pressure the child applies while writing as a means of difficulty, scientific evidence for justifying their observations was lacking. No significant differences in mean pressure applied towards the writing surface were found between children with and without handwriting difficulties (e.g., [2, 43]. The results of the current study indicate that the pressure applied to the paper, as well as pen stroke height, reflects specifically the amount of executive control which involves inhibition, planning and organization, monitoring and emotional control abilities among these children.

Dysgraphia is a learning disability subtype classified under the umbrella of *neurodevelopmental hidden disabilities* [52] and is found in high comorbidity with DCD and ADHD. Thus, it is not trivial to find and detect these children's deficient performance. The current study highlights the benefits of the HPSQ for identifying these children while the results of their EF scores and their relationships with handwriting performance have both a theoretical contribution as well as practical clinical implications. Taken together, the findings suggest that combining teacher and parent report data with objective measures of handwriting performance is indeed an ecologically valid assessment, which is sensitive to EF deficits manifested in the daily function of children with dysgraphia. In addition to using the HPSQ, a computer-based analysis of handwriting performance may be used as an objective, simple, quick, and relatively inexpensive method for evaluating handwriting proficiency among this population. Combining these two tools with the BRIEF may provide a picture of the child's activity performance (handwriting) features as well as the child's EF abilities as the underlying mechanism of actual daily performance.

Detecting EF deficits through handwriting features is important because EF components such as inhibition, emotional control, working memory and monitoring are required for varied daily functions besides handwriting. In fact, the handwriting process characteristics of these children reflect the manifestation of their EF deficits and detecting the deficits may be the first stage in addressing their needs to improve their daily function. Therefore, besides achieving better insight into the theoretical description of EF as an important possible underlying mechanism of handwriting difficulties, the results lead to the need to study whether providing children with dysgraphia with *EF strategies* may improve their performance.

In summary, this study addresses the call for the need for further knowledge about handwriting production, as this is a skill still required in schools (e.g., [53, 54]), particularly due to the implications of slow and non-proficient writing on children's academic achievements and self-esteem [55]. The relationships between EF abilities and the production of a specific activity such as handwriting which may be equivalent to production of other daily activities was demonstrated.

## Limitations and future research

This study is subject to certain limitations. Although the BRIEF [39] is a well-established evidence-based questionnaire, it is a parent report measure. In recent years, additional ecological valid measures for assessment of EF suited for children of this age have been developed (e.g., [56, 57]. Future research should incorporate such measures in order to further establish the correlations. Additionally, further studies are required to discover what brain mechanism is behind developmental dysgraphia, and what the relevance of the frontal lobes, in connection with executive control, is to the writing disability. Practically, strategies to improve working memory, inhibition, emotional control, and monitoring as related to letter creation as well as to other daily functions should be emphasized. Such strategies may lead to an improvement in handwriting performance and consequently improve academic success and self-efficacy. These strategies may also influence success in other life tasks and domains such as activities of daily living, work, leisure and social participation [58].
